# Cytoplasmic p53 aggregates accumulated in p53-mutated cancer correlate with poor prognosis

**DOI:** 10.1093/pnasnexus/pgac128

**Published:** 2022-07-25

**Authors:** Naoyuki Iwahashi, Midori Ikezaki, Yoshihiro Komohara, Yukio Fujiwara, Tomoko Noguchi, Kaho Nishioka, Kazuko Sakai, Kazuto Nishio, Mitsuharu Ueda, Yoshito Ihara, Kenji Uchimura, Kazuhiko Ino, Kazuchika Nishitsuji

**Affiliations:** Department of Obstetrics and Gynecology, School of Medicine, Wakayama Medical University, Wakayama 641-8509, Japan; Department of Biochemistry, School of Medicine, Wakayama Medical University, Wakayama 641-8509, Japan; Department of Cell Pathology, Graduate School of Medical Sciences, Kumamoto University, Kumamoto 860-8556, Japan; Department of Cell Pathology, Graduate School of Medical Sciences, Kumamoto University, Kumamoto 860-8556, Japan; Department of Obstetrics and Gynecology, School of Medicine, Wakayama Medical University, Wakayama 641-8509, Japan; Department of Obstetrics and Gynecology, School of Medicine, Wakayama Medical University, Wakayama 641-8509, Japan; Department of Genome Biology, Kindai University Faculty of Medicine, Osaka 589-8511, Japan; Department of Genome Biology, Kindai University Faculty of Medicine, Osaka 589-8511, Japan; Department of Neurology, Graduate School of Medical Sciences, Kumamoto University, Kumamoto 860-8556, Japan; Department of Biochemistry, School of Medicine, Wakayama Medical University, Wakayama 641-8509, Japan; Unité de Glycobiologie Structurale et Fonctionnelle, UMR 8576 CNRS, Université de Lille, 59655 Villeneuve d'Ascq, France; Department of Obstetrics and Gynecology, School of Medicine, Wakayama Medical University, Wakayama 641-8509, Japan; Department of Biochemistry, School of Medicine, Wakayama Medical University, Wakayama 641-8509, Japan; Department of Pathology and Laboratory Medicine, Institute of Biomedical Sciences, Tokushima University Graduate School, Tokushima 770-8503, Japan

**Keywords:** high-grade serous ovarian carcinoma, p53, amyloid, chemoresistance, cisplatin

## Abstract

Recent studies suggested that aggregates of mutant p53 proteins may propagate and impair normal p53 functioning in recipient cells. Our previous study showed that cancer cell-derived p53 aggregates that cells internalized interfered with p53-dependent apoptosis in recipient cells. However, involvement of p53 aggregate propagation in cancer pathology has not been fully elucidated. Here, we screened patients with high-grade serous ovarian carcinoma, which is characterized by an extremely high frequency of *TP53* gene mutations, to show that patients with cytoplasmic p53 deposits have a poor prognosis compared with patients with complete p53 absence or strong nuclear p53 positivity. Cytoplasmic p53 in the patients with poor prognosis consisted of protein aggregates, which suggests that p53 aggregates are oncogenic drivers. Indeed, an inhibitor of p53 aggregation restored cellular apoptosis, a proper p53 function, in p53 aggregate-bearing patient-derived tumor organoids. In cell-based assays, endogenous and exogenous mutant p53 aggregates hindered chemotherapeutic activity of cisplatin, which depends on normal p53 functions. This inhibition was reduced by blocking p53 aggregation or internalization of p53 aggregates. Our study, thus indicates the involvement of p53 aggregate transmission in poor prognosis and in chemotherapy resistance in cancers.

Significance Statement High-grade serous ovarian carcinoma (HGSOC) is characterized by an extremely high frequency of *TP53* gene mutations. We and others suggested that mutant p53 proteins form amyloid-like aggregates that accumulate in and are released from p53 mutant cancer cells. However, involvement of p53 aggregates in cancer pathology is unclear. Here, we found that HGSOC patients with cytoplasmic p53 aggregates had a poor prognosis compared with patients with aberrant p53. Resistance to platinum chemotherapy is critical for HGSOC mortality. In cell-based assays, endogenous and exogenous mutant p53 aggregates hindered chemotherapeutic activity of cisplatin by interfering with the apoptotic function of wild-type p53. Our study, thus indicates a strong connection between p53 aggregates and prediction of prognosis and chemotherapy resistance in HGSOC.

## Introduction

*TP53* mutations have been implicated in the pathogenesis of many human cancers (1). *TP53* is the gene in human cancers that is most frequently mutated, including in more than 95% of ovarian cancers (1–3). A product of the *TP53* gene, tumor protein p53, acts as a stress sensor that triggers cell cycle arrest in response to various stresses (4). This canonical p53 function has been implicated in tumor suppression by p53. In addition, recent studies suggest that p53 plays additional roles in diverse processes, such as cellular metabolism, invasion and metastasis, and cell–cell communication within the tumor microenvironment, all of which may be involved in tumor suppression (5). Cancer-associated p53 mutations result in not only loss of tumor-suppressive function, but also dominant-negative gain of oncogenic functions of the mutant p53 proteins (2, 6). Under physiological conditions, p53 proteins are proteasomally degraded via ubiquitination by the E3 ubiquitin ligase MDM2, which maintains low p53 protein levels and makes the levels undetectable by immunohistochemistry (4). In tumor cells with mutant or wild-type p53, p53 proteins often accumulate in the nucleus and the cytoplasm because of the increased half-lives of mutant p53 and a perturbed protein quality control system (7–9). Several studies documented cytoplasmic accumulation of p53 as a prognostic indicator in colorectal adenocarcinoma, but the mechanisms of this association are unclear (10–13).

High-grade serous ovarian carcinoma (HGSOC) is the most aggressive and lethal malignancy in gynecology (14). Because patients with ovarian cancer manifest no early warning symptoms and are mostly diagnosed at advanced stages, almost 80% of ovarian cancers are of the high-grade serous type, are the most histologically aggressive of ovarian cancers, and have a poor long-term prognosis (15, 16). Ovarian cancers, particularly high-grade serous types, demonstrate considerable intertumor genetic heterogeneity with features such as different copy number abnormalities and common passenger substitutions (17–19). One frequent genetic feature in HGSOC comprises mutations in the *TP53* gene (20). A previous study reported that cytoplasmic p53 immunostaining accurately predicted *TP53* mutations (21). Self-aggregation and amyloid formation of wild-type and mutant p53 proteins were originally reported in 2003 (22) and have been implicated in the pathologies of various cancers (23–27). We previously showed that p53 aggregates accumulated in the cytoplasm of ovarian cancer cells and may interfere with normal functions of wild-type p53 after internalization (28). However, the involvement of cytoplasmic accumulation of p53 aggregates in the prognosis of HGSOC remains to be clarified. In the previous study, we identified a sulfated glycosaminoglycan-mediated spreading mechanism of p53 aggregates (28). Although the peptide-based p53 aggregation inhibitor ReACp53 reportedly increased carboplatin efficacy in HGSOC (29), the contribution of transcellular spreading to resistance to platinum chemotherapy in p53-mutated cancer is yet to be elucidated. In the present study, we show that cytoplasmic accumulation of p53 aggregates correlates with worse survival rates among patients with aberrant p53 immunohistochemical staining in HGSOC. Moreover, our cell-based assays provide evidence that extracellularly released p53 aggregates interfere with the cytotoxic activity of cisplatin, a platinum-based chemotherapeutic agent often used in ovarian cancers (30), in the recipient cells. These results indicate that cell-autonomous and cell-nonautonomous functions of p53 aggregates are involved in poor prognosis and chemotherapy resistance in HGSOC.

## Materials and Methods

### Patients and samples

This study was approved by the Ethics Committee of Wakayama Medical University (authorization no. 2025) and was conducted in accordance with the tenets of the Declaration of Helsinki. We obtained written informed consent from all patients for the use of tissue samples. The current study included 121 patients with wild-type p53 or mutant p53 ovarian cancer who had undergone neoadjuvant chemotherapy (NAC) at the Wakayama Medical University Hospital between January 2015 and March 2020. Y.K., N.I., and K.I. examined hematoxylin and eosin-stained sections so as to determine the histological diagnoses of all patients according to the World Health Organization criteria. Staging of tumors were performed according to the International Federation of Gynecology and Obstetrics classification. Images obtained from computed tomography, magnetic resonance imaging, and positron emission tomography/computed tomography, and their related clinical findings, together with ascitic or pleural fluid cytological findings, were used to diagnose patients who had NAC and identify disease stages. Most patients who had NAC, except those with stage IA disease, received three to six cycles of paclitaxel (175 mg/m^2^, day 1) and carboplatin (five areas under the curve, day 1) with or without bevacizumab (15 mg/kg, day 1) every 21 days as postoperative therapy or NAC. Patients who were resistant to first-line chemotherapy received second-line regimens, which mainly included cisplatin (60 mg/m^2^, day 1) and irinotecan (60 mg/m^2^, days 1, 8, and 15). A total of 25 patients who received NAC, whose cancer cells demonstrated a marked or complete response to chemotherapy were excluded from the Kaplan–Meier analysis of survival.

### Extraction of tumor DNA and sequencing

To screen for *TP53* mutations, hematoxylin and eosin-stained formalin–fixed paraffin–embedded specimens of ovarian cancer were reviewed histologically, and those possessing at least 75% of tumor cells were used for DNA extraction. A total of 40 ng of tumor DNA for each case was subjected to targeted sequencing with the QIAseq Human Comprehensive Cancer Panel for 275 genes (Qiagen, Valencia, CA, USA). We prepared libraries according to the manufacturer’s protocol (31), after which they were pooled and sequenced by using a NextSeq 500 instrument (Illumina, San Diego, CA, USA). We aligned reads to the hg19 human reference genome and identified variants according to the manufacturer’s pipeline. We excluded germline mutations based on the Human Genetic Variation Database (https://www.hgvd.genome.med.kyoto-u.ac.jp/) and the Exome Aggregation Consortium database (32).

### Materials

Rabbit polyclonal anti-β-actin antibody was obtained from Medical and Biological Laboratories (Tokyo, Japan). Rabbit monoclonal (E26) and the mouse monoclonal (DO-1) antibodies to p53 were purchased from Abcam (Cambridge, UK). Rabbit IgG isotype control and the mouse monoclonal to p53 (DO-7) were purchased from Cell Signaling Technology (Danvers, MA, USA). The antioligomer antibody (A11) and the antiamyloid antibody (OC) were purchased from StressMarq Biosciences (Victoria, BC, Canada). ReACp53, an inhibitor of p53 aggregation (24), was obtained from Selleck Chemicals (Houston, TX, USA). pCMV-Neo-Bam and pCMV-Neo-Bam p53 R248W (Addgene plasmid # 16440, http://n2t.net/addgene:16440, RRID:Addgene_16436, and Addgene plasmid # 16437, http://n2t.net/addgene:16437, RRID:Addgene_16437) were gifts from Dr Bert Vogelstein (33).

### Cell culture

Because we and others previously showed that OVCAR-3 cells carrying the p53 R248Q mutation homeostatically produced mutant p53 aggregates and released them into culture media (28, 34), we used OVCAR-3 cells as a model for p53 aggregate-producing ovarian cancer cells. OVCAR-3 cells were provided by the RIKEN BRC through the National Bio-Resource Project of the MEXT/AMED, Japan, and were maintained in RPMI 1640 (Wako Pure Chemical Industries, Osaka, Japan) supplemented with 10% heat-inactivated fetal bovine serum (FBS; BioWest, Nuaillé, France), 100 U/ml penicillin, and 100 μg/ml streptomycin (Sigma) at 37°C in 95% air and 5% CO_2_. Wild-type p53 MCF-7 human breast cancer cells (35) and null p53 PC-3 human prostate adenocarcinoma cells (36) from the JCRB Cell Bank (Osaka, Japan) were maintained in Dulbecco’s Modified Eagle’s Medium and Kaighn’s modification of Ham’s F12 medium supplemented with 10% heat-inactivated FBS, 100 U/ml penicillin, and 100 μg/ml streptomycin, respectively.

### Analysis of patient-derived tumor organoids

Fukushima patient-derived tumor organoids (F-PDOs) derived from ovarian cancer tissues were established by the Fukushima Translational Research Project as previously described (37). We used two ovarian cancer-derived F-PDOs, ROVA002-4 with wild-type p53 and ROVA007-2 with the G199V mutant p53. F-PDOs were maintained as previously described (37). Briefly, frozen stocks of F-PDOs were restarted and passed at least twice. F-PDOs were cultured in Cancer Cell Expansion Media (Fujifilm, Tokyo, Japan) for ROVA002-4 cells or Cancer Cell Expansion Media plus (Fujifilm) for ROVA007-2 cells by using ultra-low attachment 75-cm^2^ flasks (Corning Incorporated, Corning, NY, USA) at 37°C in 95% air and 5% CO_2_. Approximately, 80% of the culture medium for each sample was replaced with fresh medium twice weekly. When F-PDOs reached the maximum saturation density, cells were passaged at a 1:2 ratio. For analysis of the cytotoxicity of ReACp53 against F-PDOs, F-PDOs were collected by centrifugation at 200 *g* for 2 min at room temperature, after which cell pellets were suspended in 15 ml of fresh culture medium. Suspensions were cultured for additional 24 h in a 75-cm^2^ flask. The F-PDOs were minced via a CellPet FT (JTEC Corporation, Osaka, Japan) set with a filter holder containing a 70-µm mesh filter. F-PDOs were suspended in fresh culture medium, diluted 10-fold, and seeded into 96-well round-bottomed, ultra-low attachment microplates (Corning Incorporated). Cell viability was assayed by measuring the amount of ATP by using the CellTiter-Glo 3D Cell Viability Assay (Promega Corporation, Madison, WI, USA) as previously described (37). For analysis of p53 aggregates in F-PDOs, we prepared paraffin-embedded cell blocks after fixing F-PDO pellets with 10% formalin for 24 h. Cell blocks were sectioned and stained with the E26 rabbit monoclonal anti-p53 antibody and ProteoStat Protein Aggregation Assay kit (Enzo Life Sciences, Farmingdale, NY, USA) as described below.

### Detection of protein aggregates in HGSOC tissues

Protein aggregates in HGSOC tissues were detected by means of the ProteoStat Protein Aggregation Assay kit according to the manufacturer’s protocol with modifications based on a previous study (38). Briefly, paraffin-embedded HGSOC tissues were cut into 3-µm-thick sections, and sections were deparaffinized with xylene and rehydrated with graded ethanol. Heat-induced antigen retrieval was performed by boiling the sections in a pressure cooker in Antigen Unmasking Solution (pH 6.0; Vector Laboratories, Burlingame, CA, USA) for 20 min. Sections were then blocked in Animal-Free Blocker (Vector Laboratories) for 1 h at room temperature and incubated with the anti-p53 antibody (E26, 1:100 in Animal-Free Blocker) overnight at 4°C. Sections were washed three times in phosphate-buffered saline (PBS) and were incubated with Alexa Fluor 488-conjugated polyclonal goat antirabbit IgG (1:500; Thermo Fisher Scientific, Waltham, MA, USA) for 30 min at room temperature. After sections were washed again with PBS, they were incubated in ProteoStat solution (1:2,000 in the ProteoStat assay buffer) for 3 min and were then destained in 1% acetic acid for 20 min at room temperature. The sections were mounted with Vectashield mounting medium with DAPI (Vector Laboratories) and were studied with an LSM700 confocal microscope (Zeiss, Oberkochen, Germany) and the ZEN 3.2 (Blue edition) software (Zeiss). Quantifying colocalization of the p53 and the ProteoStat signals in the nuclear/cytoplasmic p53-positive cases was achieved by calculating Pearson’s cross-correlation coefficients by using a MATLAB (The MathWorks, Natick, MA, USA) code (39), as described elsewhere (40, 41). A coefficient of 0 indicates no colocalization and a coefficient of 1.0 indicates complete colocalization. Fluorescence intensities of the green channel (p53) and the red channel (ProteoStat) in stained specimens of digital images were determined semiquantitatively by means of ImageJ software (National Institutes of Health, Bethesda, MD, USA). Among patients with cytoplasmic and nuclear p53 positivity, ProteoStat staining (four patients) and determination of p53 status (seven patients) were not available, possibly because samples deteriorated.

### Immunocytochemistry

OVCAR-3 cells were cultured on poly-L-lysine-coated cover glasses for 12 h and treated with ReACp53 (15 µM) for 20 h at 37°C. Cells were fixed at room temperature for 20 min in a solution of paraformaldehyde (4% in PBS). Cells were then washed three times with PBS, after which they were blocked and permeabilized with 10% normal goat serum containing 0.05% saponin in PBS for 20 min at room temperature. Cells were incubated with the rabbit monoclonal antihuman p53 antibody (E26, 1:100) followed by incubation with Alexa Fluor 488-conjugated polyclonal goat antirabbit IgG (1:500; Thermo Fisher Scientific). For ProteoStat staining, sections were washed with PBS and then incubated in ProteoStat solution (1:2,000 in the ProteoStat assay buffer) for 3 min, and then they were destained in 1% acetic acid for 20 min at room temperature. The sections were mounted with Vectashield mounting medium with DAPI. The specimens were examined under an LSM700 confocal microscope.

### Analysis of the extracellular release of mutant p53 aggregates by OVCAR-3 cells

OVCAR-3 cells were cultured in serum-free RPMI 1640 for 24 h in the presence or absence of ReACp53 (15 µM), after which the culture medium was collected. Cells and debris were removed by centrifuging the culture media at 2,000 *g* for 30 min, and thereby OVCAR-3 conditioned medium (CM) was obtained. We used OVCAR-3 CM as a p53 aggregate-containing culture medium as previously described (28). Protein aggregates in the OVCAR-3 CM were then immunoprecipitated by using the antioligomer antibody (A11, 1:50) or the antiamyloid antibody (OC, 1:50) that were conjugated with Dynabeads Protein G (Thermo Fisher Scientific). Dynabeads were collected and heated for 10 min at 70°C in sodium dodecyl sulfate–polyacrylamide gel electrophoresis (SDS–PAGE) sample buffer. The supernatants were subjected to SDS–PAGE with 5% to 20% gels (Wako Pure Chemical Industries) and were transferred to polyvinylidene difluoride (PVDF) membranes (Millipore, Billerica, MA, USA). Membranes were then incubated with the DO-1 anti-p53 monoclonal antibody (1:1,000), followed by a preabsorbed horseradish peroxidase-conjugated antimouse IgG (1:10,000; Jackson ImmunoResearch Laboratories, West Grove, PA, USA). Signals were visualized and analyzed by using a LuminoGraph image analyzer (ATTO, Tokyo, Japan).

### Analysis of cytoplasmic p53 aggregates in OVCAR-3 cells

Cytoplasmic p53 aggregates in OVCAR-3 cells were analyzed by means of Blue native PAGE (BN-PAGE). BN-PAGE was performed with the NativePAGE Bis-Tris Gel system (Thermo Fisher Scientific) according to the manufacturer’s instructions. Briefly, OVCAR-3 cells grown in 6-cm dishes were harvested and lysed by using 100 µl of the NativePAGE 1X Sample Buffer containing 0.1% digitonin (Thermo Fisher Scientific) and a protein inhibitor cocktail (Roche, Basel, Switzerland), and samples were then centrifuged at 20,000 *g* for 30 min at 4°C. Supernatants were collected and subjected to BN-PAGE by using NativePAGE 3% to 12%, Bis-Tris, 1.0 mm, Mini Protein gels (Thermo Fisher Scientific), and the NativePAGE Running Buffer Kit (Thermo Fisher Scientific). Electrophoresis was performed at 150 V for 75 min by using the Light Blue Cathode buffer and 1X NativePAGE Running Buffer (anode, Thermo Fisher Scientific) at 4°C. After electrophoresis, proteins were transferred to PVDF membranes (Millipore) and then probed with the rabbit monoclonal anti-p53 antibody (E26, 1:1,000), followed by incubation with a preabsorbed horseradish peroxidase-conjugated antirabbit IgG (1:10,000; Jackson ImmunoResearch Laboratories). Signals were visualized and analyzed by using a LuminoGraph image analyzer.

### Immunohistochemistry

Paraffin-embedded HGSOC blocks were cut into 3-μm-thick sections, after which sections were deparaffinized and rehydrated. Epitopes were then retrieved by means of heat-induced antigen retrieval—boiling the sections in a pressure cooker in citrate buffer (10 mM sodium citrate, 0.05% Tween 20, pH 6.0) for 20 min. The DO-7 mouse monoclonal anti-p53 antibody that was used for p53 immunohistochemical evaluation of ovarian carcinoma (21) was the primary antibody (1:100). Sections were then incubated with a horseradish peroxidase-labeled goat antimouse secondary antibody (Histofine; Nichirei Biosciences, Tokyo, Japan). Signals were detected by using a diaminobenzidine substrate kit (Nichirei Biosciences). A senior pathologist (Y.K.) and experienced oncologists (N.I. and K.I.) analyzed the specimens.

### Analysis of resistance to cisplatin chemotherapy

We used, in addition to OVCAR-3 CM, CM of PC-3 cells expressing the R248W p53 mutant, which we discovered in our previous study released p53 aggregates (28). pCMV-Neo-Bam or pCMV-Neo-Bam p53 R248W was delivered to p53-null PC-3 cells by using the ViaFect Transfection Reagent (Promega). Cells were cultured for 48 h and culture media were collected. Cells and debris were removed from the culture media by centrifugation at 2,000 *g* for 30 min and supernatants were then used as PC3 CM-mock and PC3 CM-R248W.

OVCAR-3 cells in 48-well plates were cultured in the presence or absence of ReACp53 (10 µM) for 3 h, after which cisplatin (Bristol–Myers Squibb Company, New York, NY, USA) was added to the culture medium to obtain a final concentration of 5 µM, and the culture continued for 17 h. Cell viability was assessed by using the Cell Counting Kit-8 (Dojindo, Kumamoto, Japan). In other experiments, OVCAR-3 cells in 12-well plates were cultured in the presence or absence of ReACp53 (10 µM) for 3 h, after which cisplatin was added to the culture medium to obtain a final concentration of 10 µM, and the culture proceeded for 17 h. Cells were then fixed with 10% trichloroacetic acid in PBS for 30 min at 4°C, and whole cell lysates were prepared by means of trichloroacetic acid precipitation as previously described (28). Activation of caspase 3 was analyzed by means of western blotting with a specific antibody against the activated (cleaved) form of caspase 3 (1:1,000; Cell Signaling Technology).

For analysis of the effect of cell uptake of extracellular p53 aggregates on wild-type p53 of recipient cells, MCF-7 cells were treated with OVCAR-3 CM, PC3 CM-mock, or PC3 CM-R248W for 8 h to allow MCF-7 cells to internalize p53 aggregates. To competitively inhibit cell uptake of p53 aggregates, heparin (5 µg/ml), which is a structural analogue of the highly sulfated domains of heparan sulfate, was used (28, 42). Cisplatin was then added to the culture medium to obtain a final concentration of 25 µM, and MCF-7 cells were cultured in that medium for 24 h. Cell viability was analyzed by using the Cell Counting Kit-8.

### Statistical analysis

Data were analyzed via an ordinary one-way analysis of variance with post hoc Dunnett or Bonferroni test by means of Prism software (GraphPad Software, Version 7.04, San Diego, CA, USA). We analyzed clinical data by means of JMP Pro (Statistical Discovery Software, Version 13.1.0, Cary, NC, USA). We compared two groups via Fisher’s exact test or the Wilcoxon test to analyze categorical variables and continuous variables, respectively. Progression-free and overall survival rates were evaluated by using the Kaplan–Meier method, and log-rank tests were used for comparisons of the groups. We used the Cox proportional hazards model to assess the relationship between clinicopathological parameters and survival. Results were said to be significant when *P-*values were less than 0.05.

## Results

### Assignment of HGSOC cases to four groups by using p53 immunohistochemistry

**Fig. 1.**
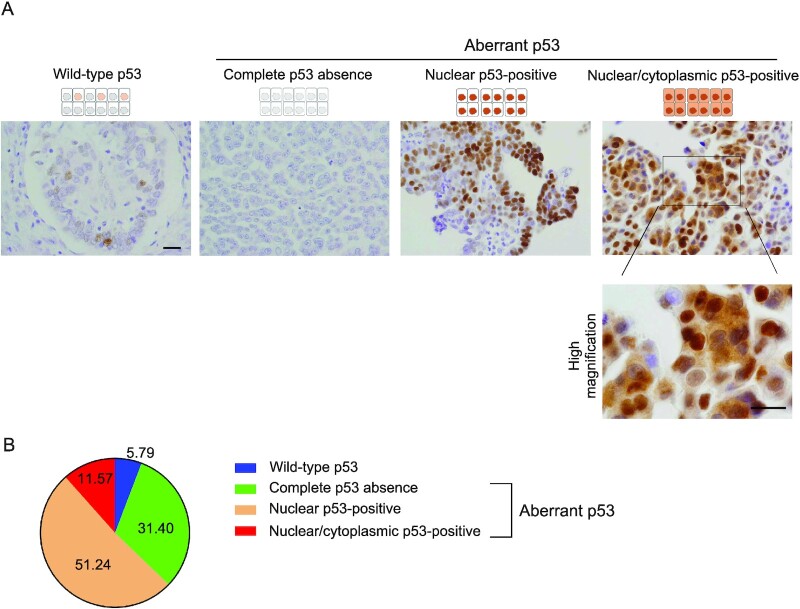
Immunohistochemical analysis of p53 in HGSOC. (A) Representative immunohistochemical images of HGSOC tissues with wild-type p53, complete p53 absence, nuclear p53-positive, and nuclear/cytoplasmic p53-positive patterns. Scale bar: 20 µm. (B) Pie chart showing the percentages of patients with HGSOC and wild-type p53, complete p53 absence, nuclear p53-positive, and nuclear/cytoplasmic p53-positive patterns. Numbers in the pie chart indicate the percentages of the different p53-staining patterns.

First, to identify HGSOC cases with large quantities of intracellular p53, we screened 121 patients with HGSOC by using immunohistochemical analysis. We used the DO-7 monoclonal anti-p53 antibody for immunohistochemical analysis of HGSOC as previously reported (21). In p53-positive cases, not all tumor cells were positive, and the signal intensities varied among tumor cells in the same section. A previous study suggested using a 3-category scoring system of p53 staining patterns of HGSOC tissues: wild-type with subtle or low nuclear staining signals, complete absence, and overexpression with strong nuclear staining signals (43). Here, we also found another staining pattern. We, thus classified the HGSOC cases into four groups: wild-type p53 cases, complete p53 absence cases, nuclear p53-positive cases (overexpression with strong nuclear staining signals), and nuclear/cytoplasmic p53-positive cases (strong nuclear and cytoplasmic staining signals). Figure 1(A) provides representative images of each case. Among the 121 cases tested, seven cases and 38 cases were found to have wild-type p53 and complete p53 absence patterns, respectively. Strong nuclear p53 staining occurred in 62 cases, and nuclear/cytoplasmic p53 staining occurred in 14 cases (Fig. 1B). We did not find any cytoplasmic-positive/nuclear-negative cases.

### Poor prognosis of patients with HGSOC and cytoplasmic p53 aggregates

**Fig. 2.**
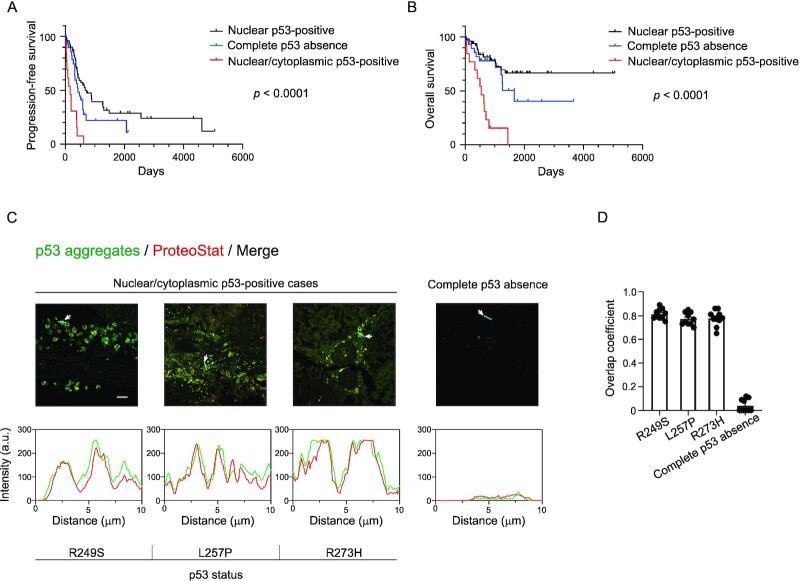
Correlation between clinical prognosis or clinical stages and immunohistochemical analysis of aberrant p53 in HGSOC and cytoplasmic p53 as p53 aggregates in nuclear/cytoplasmic p53-positive cases. Kaplan–Meier survival curves for progression-free survival rates (A) and overall survival rates (B) of patients with HGSOC and complete p53 absence, strong nuclear p53-positive, and nuclear/cytoplasmic p53-positive patterns. (C) Detection of p53 and p53 aggregates by using the E26 rabbit monoclonal anti-p53 antibody (green) and ProteoStat (red) in nuclear/cytoplasmic p53-positive HGSOC tissues. An immunohistochemical image of an HGSOC tissue with complete p53 absence is also shown. The labels at the bottom indicate the p53 status of each patient. The signal intensities along the line markers (blue lines) indicated by arrows in the images were measured for the green channel (p53) and the red channel (ProteoStat). Scale bar: 20 µm. (D) Quantification of colocalization of the green channel (p53) and the red channel (ProteoStat) obtained with Pearson’s cross-correlation coefficient analysis. Pearson’s cross-correlation coefficients were determined in 10 randomly selected regions of interest (20 µm × 20 µm) for each specimen.

We and other groups showed that mutant p53 forms amyloid-like aggregates that may contribute to cancer development (6, 28, 44). We next investigated the survival rates of three aberrant p53 HGSOC groups. We excluded patients from the Kaplan–Meier analysis study if they had NAC and their cancer cells demonstrated a marked or complete response to chemotherapy. Table S1 (Supplementary Material) provides a summary of the clinical details that were used for Kaplan–Meier analysis. This analysis indicated that patients with nuclear/cytoplasmic p53 staining signals had worse progression-free survival and overall survival rates compared with patients with complete p53 absence or strong p53 nuclear signals (Fig. 2A and B).

**Fig. 3.**
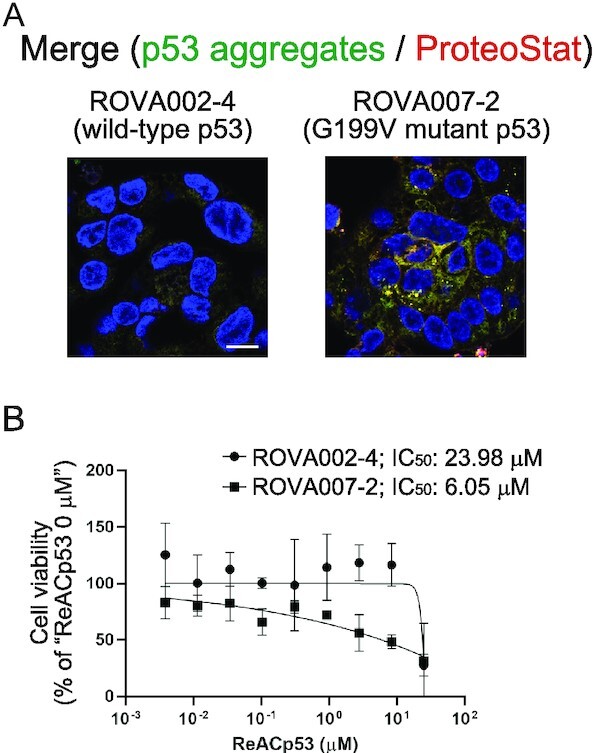
Disaggregating p53 aggregates by ReACp53 caused cell death in PDOs. (A) Detection of p53 and p53 aggregates by using the E26 rabbit monoclonal anti-p53 antibody (green) and ProteoStat (red) in two F-PDOs, ROVA002-4 with wild-type p53 and ROVA007-2 with the G199V mutant p53. Scale bar: 10 µm. (B) Dose–response curves of F-PDOs ReACp53. The F-PDOs were minced, seeded in 96-well plates, and treated with eight different concentrations of ReACp53 for 8 days. Cell viabilities were analyzed by measuring the ATP levels in each well. The data represent the mean ± standard deviation of three experiments.

The aim of the current study was to investigate the contribution of p53 aggregates to cancer pathology. We previously showed that cytoplasmic p53 deposits in HGSOC tissues contained highly sulfated domains of heparan sulfate (28), which are common components of tissue amyloid deposits in almost all amyloidoses (45). We hypothesized that the cytoplasmic p53 in patients with HGSOC and poor prognosis is an aggregated form of p53. We, thus investigated whether p53 mutants in tumors of the nuclear/cytoplasmic p53-positive samples formed p53 aggregates. We used the ProteoStat protein aggregation detection dye (38, 46), which is utilized to find protein aggregates in mammalian tissues. We found cytoplasmic accumulation of ProteoStat-positive p53 aggregates in 10 nuclear/cytoplasmic p53-positive cases (Fig. 2C; Figure S1B, Supplementary Material). Figure 2(C) shows three representative immunohistochemical results of these positive cases. Quantitative analysis of the colocalization between p53 and protein aggregates (ProteoStat) was also performed by determining Pearson’s colocalization coefficients. Both Pearson’s colocalization coefficients and analysis of fluorescent intensities supported the finding that cytoplasmic protein aggregates are made of p53 (Fig. 2C and D; Figure S1B and C, Supplementary Material). We previously reported that mutant p53 aggregates hindered the apoptotic function of wild-type p53 in recipient cells (28). Here, we confirmed this by using patient-derived tumor organoids (PDOs). We used two F-PDOs, ROVA002-4 expressing wild-type p53 and ROVA007-2 expressing the G199V mutant p53. The ROVA007-2 PDO, but not the ROVA002-4 PDO, contained aggregated p53 in the cytoplasm (Fig. 3A). Soragni et al. (24) reported a cell-penetrating inhibitor of p53 aggregation, ReACp53. Treating these F-PDOs with ReACp53 reduced cell viability in a dose-dependent manner (Fig. 3B). Notably, the IC_50_ of ReACp53 in the ROVA002-4 PDO was four times higher than that in the ROVA007-2 PDO, which contains p53 aggregates. These results supported the result that ReACp53 restored the proper p53 functions in p53 aggregate-bearing PDOs (24).

### Elimination of p53 aggregates from cytoplasm and CM by ReACp53

**Fig. 4.**
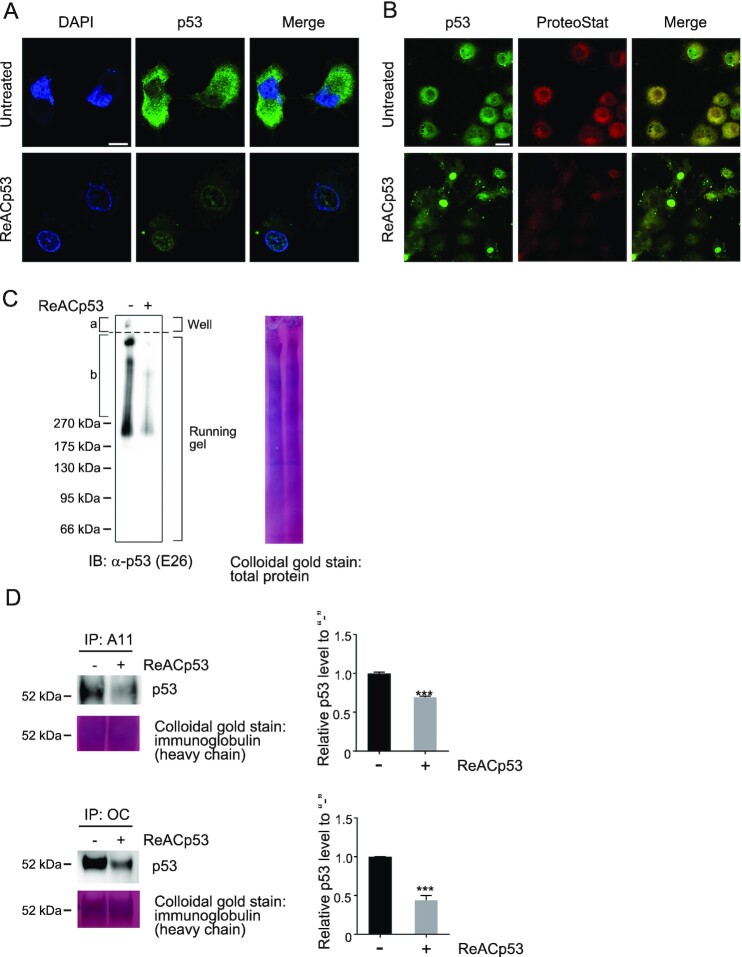
Disaggregation of p53 aggregates in OVCAR-3 cells. (A) The ReACp53 p53 aggregate inhibitor reduced the amount of p53 staining and redistributed p53 to the nucleus in OVCAR-3 cells. OVCAR-3 cells were treated with the ReACp53 p53 aggregate inhibitor (15 µM) for 20 h at 37°C. Cells were then stained with the E26 rabbit monoclonal anti-p53 antibody and counterstained with DAPI. Scale bar: 10 µm. (B) and (C) ReACp53 reduced the amount of p53 aggregates in OVCAR-3 cells. (B) OVCAR-3 cells were treated with the ReACp53 p53 aggregate inhibitor (15 µM) for 20 h at 37°C. p53 aggregates were stained with the ProteoStat protein aggregate detection dye and the E26 rabbit monoclonal anti-p53 antibody. Scale bar: 20 µm. (C) Cytosolic p53 aggregates were analyzed by means of BN-PAGE followed by western blotting. OVCAR-3 cells were treated with the ReACp53 p53 aggregate inhibitor (15 µM) for 20 h at 37°C, after which cells were lysed and separated by using BN-PAGE. Proteins were then transferred to PVDF membranes, and p53 aggregates were probed with the E26 anit-p53 antibody. Large amyloid fibrils that did not enter the running gel (a) as well as many aggregated species with various molecular weights were observed (b). After p53 amyloid was detected, the membranes were stained with colloidal gold. (D) ReACp53 reduced the extracellular release of p53 aggregates by OVCAR-3 cells. OVCAR-3 cells were treated with the ReACp53 p53 aggregate inhibitor (15 µM in serum-free Opti-MEM) for 20 h at 37°C. To detect p53 amyloid in conditioned media, amyloid in the media samples were immunoprecipitated with the antioligomer A11 antibody or the antiamyloid fibril OC antibody, and the immunoprecipitates were subjected to western blotting with the DO-1 anti-p53 monoclonal antibody. After p53 amyloid was detected, the membranes were stained with colloidal gold. The graphs show relative p53 levels. ***, *P* < 0.001 by the unpaired t test.

Here, we confirmed that ReACp53 treatment (15 µM) of OVCAR-3 cells sufficiently induced nuclear relocalization of p53 and reduced the amounts of p53 aggregates (Fig. 4A and B). Because protein aggregates may disassociate during sample preparation or electrophoresis that is used in the conventional SDS–PAGE technique, we utilized BN-PAGE followed by immunoblotting with the E26 anti-p53 antibody to visualize cytosolic p53 aggregates in OVCAR-3 cells. OVCAR-3 cells contained high-molecular-weight p53 fibrils that did not enter the running gel, and many amyloid or oligomeric assemblies had various molecular weights (Fig. 4C). We then confirmed that these p53 aggregates were reduced by treatment with ReACp53 (15 µM). Melo dos Santos et al. (47) reported that the p53 isoform Δ40p53 is a major component of cytosolic p53 aggregates in endometrial carcinoma-derived cells. Our RT-qPCR and western blotting analyses of p53 isoform expressions revealed that Δ40p53 was also expressed in OVCAR-3 cells (Figure S2A and B, Supplementary Material). In addition, we confirmed that ReACp53 treatment significantly reduced E26- or pAb1801-positive p53 by 30% to 40% (Figure S2B, Supplementary Material) and reduced pAb1801-positive p53 cytosolic aggregates (Figure S2C, Supplementary Material) in OVCAR-3 cells. We previously reported that OVCAR-3 cells released OC-positive amyloid-like p53 aggregates into culture medium (28). By using the antioligomer A11 antibody and the antiamyloid OC antibody (48, 49), we also showed here that ReACp53 treatment (15 µM) significantly reduced the extracellular release of p53 aggregates by OVCAR-3 cells (Fig. 4D).

### Cisplatin resistance caused by mutant p53 aggregates

**Fig. 5.**
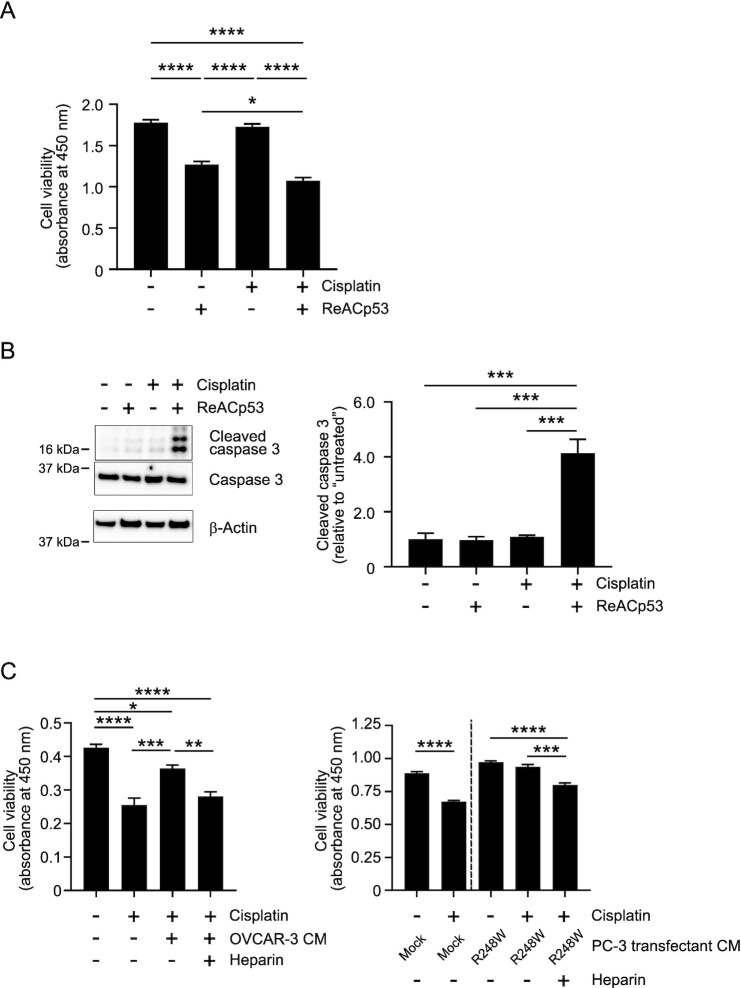
Contribution of intracellular and extracellular p53 aggregates to cisplatin desensitization. (A) and (B) Disaggregation of intracellular p53 aggregates by ReACp53 in OVCAR-3 cells enhanced cisplatin cytotoxicity. OVCAR-3 cells were treated with the ReACp53 p53 aggregate inhibitor (10 µM) and cisplatin (5 µM) for 20 h at 37°C. Cell viability was then analyzed by means of the WST-8 assay (A). Induction of apoptosis by cisplatin was analyzed by means of western blotting with an anticleaved caspase 3 antibody (B). β-Actin was used as the loading control. (C) Cell uptake of p53 aggregates desensitized recipient wild-type p53 cells to cisplatin. OVCAR-3 cells were cultured in serum-free Opti-MEM for 24 h, after which culture medium was collected. After debris was removed, the supernatant was used as p53 aggregate-containing culture medium (OVCAR-3 CM). Heparin was used as a competitive inhibitor of p53 aggregates in cell uptake. MCF-7 cells expressing wild-type p53 were pretreated with OVCAR-3 CM and heparin (5 µg/ml) for 8 h at 37°C, followed by treatment with cisplatin (25 µM) for 24 h at 37°C, after which cell viability was analyzed by means of the WST-8 assay (left). Samples of CM collected by culturing the null p53 PC-3 cells transfected with the R248W mutant p53 were also used as p53 aggregate-containing culture medium. MCF-7 cells were treated with PC-3 transfectant CM, cisplatin (25 µM), and heparin (5 µg/ml) for 30 h at 37°C, and then cell viability was analyzed by means of the WST-8 assay (right). The results showed that internalizing p53 aggregates desensitized recipient cells to cisplatin, and inhibiting cell uptake of p53 aggregates prevented the desensitization. *, *P* < 0.05; **, *P* < 0.01; ***, *P* < 0.001; and ****, *P* < 0.0001.

Platinum-based chemotherapy remains the standard of care for HGSOC (50). The cytotoxic effects of platinum agents are related to DNA damage and subsequent functional p53 activation (30), and ReACp53 reportedly had a synergistic effect by combining with carboplatin (29). Because how transmission of p53 aggregates affects neighboring cells is yet to be elucidated, we next investigated whether p53 aggregates would confer cisplatin resistance in wild-type p53-expressing recipient cells. ReACp53 treatment (10 µM) enhanced cisplatin cytotoxicity against OVCAR-3 cells by 40% (Fig. 5A). Although treatment of OVCAR-3 cells with 5 µM cisplatin induced no caspase 3 activation, treatment with both cisplatin (5 µM) and ReACp53 (10 µM) significantly enhanced caspase 3 activation (Fig. 5B), which suggested that disaggregating mutant p53 aggregates in OVCAR-3 cells restored p53 function and induced caspase 3 activation and subsequent cell death. We previously showed that extracellularly released mutant p53 aggregates impaired the functioning of p53 in the recipient cells (28, 51). Therefore, we hypothesized that cancer cell-derived p53 aggregates would contribute to cisplatin resistance in neighboring recipient cells. We used OVCAR-3 cells that release mutant p53 aggregates (28) and MCF-7 cells whose p53 status is wild-type (35). Cisplatin reduced the viability of these MCF-7 cells by 40%, but this reduction was counteracted by OVCAR-3-derived p53 aggregates (Fig. 5C). We previously showed that the highly sulfated domains of heparan sulfate mediated the cell uptake of cancer cell-derived p53 aggregates and that heparin, a structural analogue of highly sulfated domains of heparan sulfate, competitively inhibited cell uptake of p53 aggregates. Here, heparin increased the reduced cisplatin cytotoxicity by OVCAR-3-derived p53 aggregates (Fig. 5C, left). Transfection of null p53 PC-3 cells with the R248W p53 mutant-expressing plasmid (33) resulted in extracellular release of A11- and OC-positive mutant p53 aggregates [PC3 CM-R248W; Figure S3, Supplementary Material; (28)]. The reduction of the cisplatin cytotoxicity by PC3 CM-R248W was restored by adding heparin (Fig. 5C, right), which suggested that mutant p53 aggregates that were internalized by MCF-7cells interfered with the cell death function of wild-type p53 in the recipient cells.

## Discussion

Cytoplasmic staining of p53 was reportedly associated with poor prognosis in colorectal adenocarcinoma (8, 10, 11) and was suggested to be an indicator of prognosis (8). In another study, cytoplasmic p53 was detected only in small-cell lung carcinoma with an especially poor prognosis (52), which also suggested that cytoplasmic p53, but not nuclear p53, may have an increased association with prognosis. The p53 immunohistochemical assay has been proposed as serving as a surrogate for determining *TP53* mutation status in HGSOC (21, 53). In the study by Kobel et al. (21), cytoplasmic staining of p53 without nuclear overexpression was observed in four cases, but the clinical prognosis of these cases was not noted. Furthermore, in these studies, the involvement of cytoplasmic p53 aggregates was not described. In the present study, we found that HGSOC cases with accumulation of cytoplasmic p53 aggregates demonstrated significantly poor clinical survival compared with complete p53 absence cases or nuclear p53-overexpressing cases, which supported the hypothesis that cytoplasmic p53 staining may serve as an indicator of poor prognosis in HGSOC. The previous study by Kobel et al. (21) suggested that p53 mutations in cases with cytoplasmic p53 accumulation were localized in the nuclear localization signaling domain at the C-terminus to prevent nuclear localization of p53. Although cytoplasmic accumulation of wild-type p53 was found in several tumors (54, 55), in the present study we detected p53 mutations in all cases with cytoplasmic p53 proteins. In two cases (Table S2, Supplementary Material), the p53 mutations were p.E349* and p.E349fs, which are located in the nuclear localization signaling domain (56). However, in other cases, the mutations were outside the nuclear localization signaling domain. These results suggested that mechanisms other than nuclear exclusion, such as p53 aggregation, are involved in the cytoplasmic accumulation of p53 in HGSOC. Indeed, increased aggregation propensity, not subcellular localization, has been proposed as critical for the dominant-negative effects of p53 mutations (6). Several studies reported that p53 forms amyloid-like aggregates in human cancer tissues and in vitro (25, 57–59). We previously showed that mutant p53 codeposited in ovarian cancer tissues with heparan sulfate, a common nonprotein component of amyloid deposits in almost all amyloidoses (28, 45), and we suggested that p53 aggregates in mutant p53 cancers share features of amyloid fibrils. p53 mutants are often more stable compared with wild-type p53, which is generally short-lived (2), and they may lead to increases in p53 protein levels and favor p53 aggregation. p53 mutants can be divided into two categories: contact mutants with reduced DNA-binding ability and structural mutants with destabilized and unfolded structures, but the separation between these categories is not absolute (60–62). In the current study, we observed no clear correlation between the p53 status and p53 aggregate formation in HGSOC cases. In addition to the p53 status, other factors, such as sulfated glycosaminoglycans as we previously showed (28), may be involved in aggregation of p53 in vivo. Determining the mechanism of p53 aggregation in HGSOC clearly deserves additional investigation.

Tumor-associated alterations in p53 frequently result in missense mutations and lead to single amino acid substations in the p53 protein. Although these substitutions occur throughout the p53 protein, the most common cluster was found within the DNA-binding region of p53, and six “hotspot” amino acids including R175, G245, R248, R249, R273, and R282 have been identified. Given that these hotspots are located within the DNA-binding domain, alterations in the DNA-binding activity have been suggested to be critical for mutant p53 activity. p53 mutants, however, can be largely divided into two categories: structural mutants that can cause unfolding of the p53 protein, and DNA contact mutants that change amino acids critical for DNA binding (63). For example, the R175H mutant is a structural mutant and highly unfolded under physiological conditions (64), whereas the R248Q mutant is categorized as a classical contact mutant but less folded compared with the wild-type. Here, we identified a R273H case in HGSOC patients carrying cytosolic p53 aggregates. Furthermore, OVCAR-3 cells that express the R248Q mutant and PC-3 cells that express the R248W mutant released p53 aggregates that affected cisplatin activity. These results suggest that formation and spreading of p53 aggregates at least contribute to platinum resistance of the hotspot mutants that carry p53 aggregates. Our results also suggest that whether the p53 aggregate spreading-mediated chemoresistance can apply to p53 mutants including hotspot mutants would depend on the formation and release of p53 aggregates rather than the type of the mutation.

Here, our immunohistochemical approach may help identify HGSOC cases that are associated with p53 aggregates, and these cases had a worse prognosis compared with other p53 aberrant cases, which suggested that classification of p53-mutated cancers including HGSOC that is based on p53 staining patterns may be useful for distinguishing p53 aggregate-positive cases, and thereby predicting chemoresistance. In the present study, we observed large p53 amyloid aggregates and many p53 aggregates species with various molecular weights in OVCAR-3 cells. Because we currently have access only to formalin-fixed paraffin-embedded sections or PDO sections, biochemical analysis of cytoplasmic p53 aggregates in cancer tissues and PDOs is not possible. Additional analysis of cytoplasmic p53 aggregates in cancer tissues by using biochemical techniques and electron microscopy is a future challenge.

In the present study, we used mutant p53-expressing OVCAR-3 cells as an in vitro model of ovarian cancer cells with cytoplasmic p53 aggregates. In agreement with a previous study, we detected OC-positive p53 amyloid in OVCAR-3 cells (34). The p53 amyloid fibrils were reduced by treatment with ReACp53, a p53 aggregation inhibitor (24), and the results of our cell-based assays here clearly supported this finding. Intracellular p53 amyloid may interfere with the functions of monomeric p53. In our previous study, we suggested that extracellularly released mutant p53 aggregates impaired the apoptotic function of wild-type p53 in recipient cells (28, 51). Ovarian cancers including HGSOC are often refractory to platinum-based chemotherapies such as those containing cisplatin (65). However, the molecular mechanisms of this cisplatin resistance have not been well-studied (66). The cytotoxicity of platinum drugs is at least partly mediated by p53, which suggests that sensitivity to platinum drugs depends on the existence of native p53 proteins. Here, we showed that disaggregating p53 amyloid in OVCAR-3 cells resensitized OVCAR-3 cells to cisplatin. By using heparin as a competitive inhibitor of the cell uptake of p53 aggregates, we also showed that extracellularly released p53 aggregates desensitized recipient cells with wild-type p53 to cisplatin and that inhibiting cell uptake of p53 aggregates prevented this desensitization. These results clearly support the proposal that transmission of cancer cell-derived p53 aggregates at least partly contributes to platinum resistance in HGSOC. A major factor causing the loss of apoptotic function in many cancer cells is p53 mutation (67), and this loss of apoptotic function will contribute to cisplatin resistance in mutant p53 cancers (30). Several tumors or tumor models, however, demonstrated sensitivity or resistance to cisplatin regardless of *TP53* mutation (68, 69). Our results showing that intracellular and extracellular p53 aggregates conferred cisplatin resistance to the cells with p53 mutants and neighboring recipient cells may help resolve these discrepancies.

The prognostic significance of cytoplasmic p53 accumulation in HGSOC seems to have been overlooked. Many HGSOC tumors harbor at least one wild-type allele of p53, and wild-type p53 can be inactivated by incorporation into mutant p53 aggregates (6, 28, 59). Thus, our results emphasize the importance of the increased p53 aggregation tendency that may be caused by mutations of p53 proteins independently of sequence alterations resulting from mutations. In our previous study, we demonstrated that p53 aggregates that were released from cancer cells interfered with the normal functioning of p53 in recipient cells (28). These lines of evidence clearly support the importance of p53 aggregation in the pathology of mutant p53 cancers including HGSOC and resistance to platinum-based chemotherapy, and they suggest different roles of p53 aggregation: a dominant-negative function that inactivates wild-type p53 and a gain of oncogenic function. Additional studies to address the possibility of p53 aggregates as being a therapeutic target of HGSOC are warranted.

Although the involvement of intratumor p53 aggregates in cancer therapy was proposed in several reports (24, 29), cell-nonautonomous effects of p53 aggregates on chemoresistance have not been investigated. Together with our previous study that proposed the sulfated glycosaminoglycan-mediated spreading of p53 aggregates, the current study clearly demonstrated that patients with HGSOC and p53 aggregates had a poor prognosis and that cell autonomous and cell-nonautonomous p53 aggregates at least partly contributed to reduced cisplatin sensitivity in cell-based assays. The involvement of p53 aggregates in platinum resistance in HGSOC in vivo deserves additional investigation.

## Supplementary Material

pgac128_Supplemental_File

## Data Availability

All data is included in the manuscript and/or supporting information.
