# Supplementary material for: Cytoplasmic p53 aggregates accumulated in p53-mutated cancer correlate with poor prognosis
Source: PNAS Nexus. 2022 Jul 25;1(3):pgac128. doi: 10.1093/pnasnexus/pgac128 (PMC9896898; doi:10.1093/pnasnexus/pgac128)
Supplement: pgac128_Supplemental_File [file pgac128_supplemental_file.docx]

**Supplemental Materials**

**Cytoplasmic p53 aggregates accumulated in p53-mutated cancer correlate with poor prognosis**

**Naoyuki Iwahashi, Midori Ikezaki, Yoshihiro Komohara, Yukio Fujiwara, Tomoko Noguchi, Kaho Nishioka, Kazuko Sakai, Kazuto Nishio, Mitsuharu Ueda, Yoshito Ihara, Kenji Uchimura, Kazuhiko Ino, and Kazuchika Nishitsuji^*^**

^*^Correspondence: Kazuchika Nishitsuji, Ph.D., Department of Biochemistry, School of Medicine, Wakayama Medical University, 811-1 Kimiidera, Wakayama 641-8509, Japan, E-mail: [nishit@wakayama-med.ac.jp](mailto:nishit@wakayama-med.ac.jp); Phone/fax: +81-73-441-0628

**Supplementary Methods**

**Immunohistochemistry**

Paraffin-embedded HGSOC blocks were cut into 3-μm-thick sections, after which sections were deparaffinized and rehydrated. Epitopes were then retrieved by means of heat-induced antigen retrieval—boiling the sections in a pressure cooker in citrate buffer (10 mM sodium citrate, 0.05% Tween 20, pH 6.0) for 20 min. The DO-7 mouse monoclonal anti-p53 antibody that was used for p53 immunohistochemical evaluation of ovarian carcinoma (1) was the primary antibody (1:100). Sections were then incubated with a horseradish peroxidase-labeled goat anti-mouse secondary antibody (Histofine; Nichirei Biosciences, Tokyo, Japan). Signals were detected by using a diaminobenzidine substrate kit (Nichirei Biosciences). A senior pathologist (Y.K.) and experienced oncologists (N.I. and K.I.) analyzed the specimens.

**Detection of protein aggregates in HGSOC tissues**

Protein aggregates in HGSOC tissues were detected by means of the ProteoStat Protein Aggregation Assay kit according to the manufacturer’s protocol with modifications based on a previous study (2). Briefly, paraffin-embedded HGSOC tissues were cut into 3-µm-thick sections, and sections were deparaffinized with xylene and rehydrated with graded ethanol. Heat-induced antigen retrieval was performed by boiling the sections in a pressure cooker in Antigen Unmasking Solution (pH 6.0; Vector Laboratories, Burlingame, CA, USA) for 20 min. Sections were then blocked in Animal-Free Blocker (Vector Laboratories) for 1 h at room temperature and incubated with the anti-p53 antibody (E26, 1:100 in Animal-Free Blocker) overnight at 4°C. Sections were washed three times in phosphate-buffered saline (PBS) and were incubated with Alexa Fluor 488 -conjugated polyclonal goat anti-rabbit IgG (1:500; Thermo Fisher Scientific, Waltham, USA) for 30 min at room temperature. After sections were washed again with PBS, they were incubated in ProteoStat solution (1:2,000 in the ProteoStat assay buffer) for 3 min and were then destained in 1% acetic acid for 20 min at room temperature. The sections were mounted with Vectashield mounting medium with DAPI (Vector Laboratories) and were studied with an LSM700 confocal microscope (Zeiss, Oberkochen, Germany) and the ZEN 3.2 (Blue edition) software (Zeiss). Quantifying colocalization of the p53 and the ProteoStat signals in the nuclear/cytoplasmic p53-positive cases was achieved by calculating Pearson’s cross-correlation coefficients by using a MATLAB (The MathWorks, Natick, MA, USA) code (3), as described elsewhere (4, 5). A coefficient of 0 indicates no colocalization and a coefficient of 1.0 indicates complete colocalization. Fluorescence intensities of the green channel (p53) and the red channel (ProteoStat) in stained specimens of digital images were determined semiquantitatively by means of ImageJ software (National Institutes of Health, Bethesda, MD, USA). Among patients with cytoplasmic and nuclear p53 positivity, ProteoStat staining (four patients) and determination of p53 status (seven patients) were not available, possibly because samples deteriorated.

**Analysis of p53 isoforms by using RT-qPCR and Western blotting**

Transcript levels of each p53 isoform were determined by using RT-qPCR. TRIzol reagent (Thermo Fisher Scientific) was used for the extraction of total RNAs. RT-qPCR was performed with the CFX96 Touch Real-Time System (Bio-Rad Laboratories, Hercules, CA, USA) and iTaq Universal SYBR Green One-Step Kit (Bio-Rad Laboratories). Reverse transcription was performed with 0.1 µg of total RNA extracts at 50°C for 10 min and then at 95°C for 1 min. Primer sequences used were based on a previous report (6) and were as follows: p53α/(fl-p53), forward: 5´-ATG GAG CAG CCG CAG TCA GAT-3´, reverse: 5´-AAT GTC AGT CAG GCC CTT CTG TC-3´; p53β, forward: 5´-GCG AGC ACT GCC CAA CA-3´, reverse: 5´-GAA AGC TGG TCT GGT CCT GAA-3´; p53γ, forward: 5´-ACT AAG CGA GCA CTG CCC AA-3´, reverse: 5´-GTA AGT CAA GTA GCA TCT GAA GGG TG-3´; Δ40p53, forward: 5´-TCC CTG GAT TGG CAG CC-3´, reverse: 5´-TGG TGG GCC TGC CCT T-3´; Δ133p53, forward: 5´-TGA CTT TCA ACT CTG TCT CCT TCC T-3´, reverse: 5´-GGC CAG ACC ATC GCT ATC TG-3´; and GAPDH, forward: 5´-GAG TCA ACG GAT TTG GTC GT-3´, reverse: 5´-GAC AAG CTT CCC GTT CTC AG-3´. Conditions of PCR reactions for analyses of p53 isoforms were as follows: initial incubation at 50°C for 2 min, followed by 94°C for 3 min and 34 cycles at 94°C for 1 min, 58°C for 1 min, and 72°C for 1 min; followed by a final incubation at 72°C for 10 min (6). Alternatively, PCR was run for 40 cycles at 95°C for 10 s and 56°C for 30 s (GAPDH). We processed data by means of the comparative ΔΔCt method by using the Bio-Rad CFX Manager version 3.1 (Bio-Rad Laboratories). GAPDH served as the reference RNA. For analysis of p53 isoforms by Western blotting, proteins in OVCAR-3 cells were precipitated with 10%TCA in PBS for 30 min at 4°C and collected by centrifugation at 10,000 *g* for 1 h at 4°C. Proteins were lysed with the SDS/PAGE sample buffer, and p53 protein levels in samples were analyzed by means of Western blotting with the E26 rabbit monoclonal anti-p53 antibody (Abcam, Cambridge, UK, 1:1,000) and the pAb1801 mouse monoclonal anti-p53 antibody (Abcam, 1:500). Membranes were then treated with a preabsorbed horseradish peroxidase-conjugated anti-rabbit or mouse IgG (1:10,000; Jackson ImmunoResearch Laboratories, West Grove, PA, USA). Signals were visualized and analyzed by using a LuminoGraph image analyzer (ATTO, Tokyo, Japan).

**Analysis of cytoplasmic p53 aggregates in OVCAR-3 cells**

Cytoplasmic p53 aggregates in OVCAR-3 cells were analyzed by means of Blue native PAGE (BN-PAGE). BN-PAGE was performed with the NativePAGE Bis-Tris Gel system (Thermo Fisher Scientific) according to the manufacturer’s instructions. Briefly, OVCAR-3 cells grown in 6-cm dishes were harvested and lysed by using 100 µl of the NativePAGE 1X Sample Buffer containing 0.1% digitonin (Thermo Fisher Scientific) and a protein inhibitor cocktail (Roche, Basel, Switzerland), and samples were then centrifuged at 20,000 *g* for 30 min at 4°C. Supernatants were collected and subjected to BN-PAGE by using NativePAGE 3 to 12%, Bis-Tris, 1.0 mm, Mini Protein gels (Thermo Fisher Scientific) and the NativePAGE Running Buffer Kit (Thermo Fisher Scientific). Electrophoresis was performed at 150 V for 75 min by using the Light Blue Cathode buffer and 1X NativePAGE Running Buffer (anode, Thermo Fisher Scientific) at 4°C. After electrophoresis, proteins were transferred to PVDF membranes (Millipore) and then probed with the rabbit monoclonal anti-p53 antibody (E26, 1:1,000), followed by incubation with a preabsorbed horseradish peroxidase-conjugated anti-rabbit IgG (1:10,000; Jackson ImmunoResearch Laboratories). Signals were visualized and analyzed by using a LuminoGraph image analyzer.

**Analysis of Extracellular Release of p53 aggregates by PC-3 cells**

For analysis of extracellular release of p53 aggregates, p53-null PC-3 cells were transfected with pCMV-Neo-Bam or pCMV-Neo-Bam p53 R248W by using the ViaFect transfection reagent (Promega, Madison, WI). After 48 h, conditioned media samples were collected and centrifuged at 2,000 *g* for 30 min to remove debris. Conditioned media samples were then concentrated with Amicon Ultra Filters (Merck Millipore, Burlington, MA), and protein aggregates in conditioned media were immunoprecipitated by using the anti-oligomer A11 antibody (StressMarq Biosciences, Victoria, BC, 1:50) or the anti-amyloid OC antibody (StressMarq Biosciences, 1:50) and Dynabeads Protein G (Thermo Fisher Scientific). The beads were heated at 70°C for 10 min in SDS-PAGE sample buffer, and the p53 proteins in the supernatants were detected by means of Western blotting with 5–20% gradient gels and the DO-1 anti-p53 monoclonal antibody (Abcam, 1:1,000), and a preabsorbed horseradish peroxidase-conjugated anti-mouse IgG (1:10,000, Jackson ImmunoResearch Laboratories). Signals were visualized and analyzed by using the LuminoGraph image analyzer (ATTO).

| **Supplemental Table S1: Clinicopathological characteristics of patients with aberrant p53 high-grade serous ovarian carcinoma** | | | | | | | | |
| --- | --- | --- | --- | --- | --- | --- | --- | --- |
|  | | p53 N+  (n = 54) | | p53 CA  (n = 28) | | p53 N+C+  (n =14) | | *p* value |
| Age, years, mean ± SD | | 61.4 ± 10.6 | | 61.6 ± 11.8 | | 63.8 ± 12.8 | | 0.843 |
| BMI, kg/m^2^, mean ± SD | | 22.8 ± 4.7 | | 22.1 ± 3.5 | | 22.5 ± 5.0 | | 0.153 |
| Stage, n (%) | |  | |  | |  | | 0.185 |
|  | I | 2 | (3.7) | 1 | (3.6) | 1 | (7.1) |  |
|  | II | 5 | (9.3) | 2 | (7.1) | 0 | (0.0) |  |
|  | III | 38 | (70.4) | 20 | (71.4) | 11 | (78.6) |  |
|  | IV | 9 | (16.7) | 5 | (17.9) | 2 | (14.3) |  |
| Recurrence or progression, n (%) | | 39 | (72.2) | 18 | (64.3) | 13 | (92.9) | 0.143 |
|  | Platinum sensitive, n (%) | 20 | (51.3) | 8 | (44.4) | 4 | (30.8) | 0.434 |
|  | Platinum resistant, n (%) | 19 | (48.7) | 10 | (55.6) | 9 | (69.2) |  |
| Treatment, n (%) | | | | | | | | |
|  | Surgery alone | 2 | (3.7) | 2 | (7.2) | 3 | (21.4) | 0.213 |
|  | Surgery plus NAC | 25 | (46.3) | 10 | (35.7) | 5 | (35.7) |  |
|  | Surgery plus adjuvant chemotherapy | 27 | (50.0) | 16 | (57.1) | 6 | (42.9) |  |
| PFS, days, median (range) | | 560 | (30-5055) | 421 | (16-2124) | 144 | (17-623) | 0.009* |
| OS, days, median (range) | | 946 | (30-5055) | 779 | (16-3662) | 522 | (17-1439) | 0.024* |
| *, Statistically significant; *p53 N+*, nuclear p53-positive; *p53 CA,* complete p53 absence; *p53 N+C+,* nuclear/cytoplasmic p53-positive; *BMI,* body mass index; *OS,* overall survival; *PFS,* progression-free survival | | | | | | | | |

**Supplemental Table S2: Status of p53 mutants for nuclear/cytoplasmic p53-positive cases**

| Patient ID | p53 |
| --- | --- |
| 2065 | p.R249S |
| 3281 | p.V157F |
| 6549 | p.R273H |
| 11494 | p.E349fs |
| 6194 | p.Y205C |
| 10916 | p.E349* |
| 4291 | p.L257P |
| 8105 | N.D. |
| 8204 | N.D. |
| 9491 | N.D. |

*N.D.,* *not determined.*


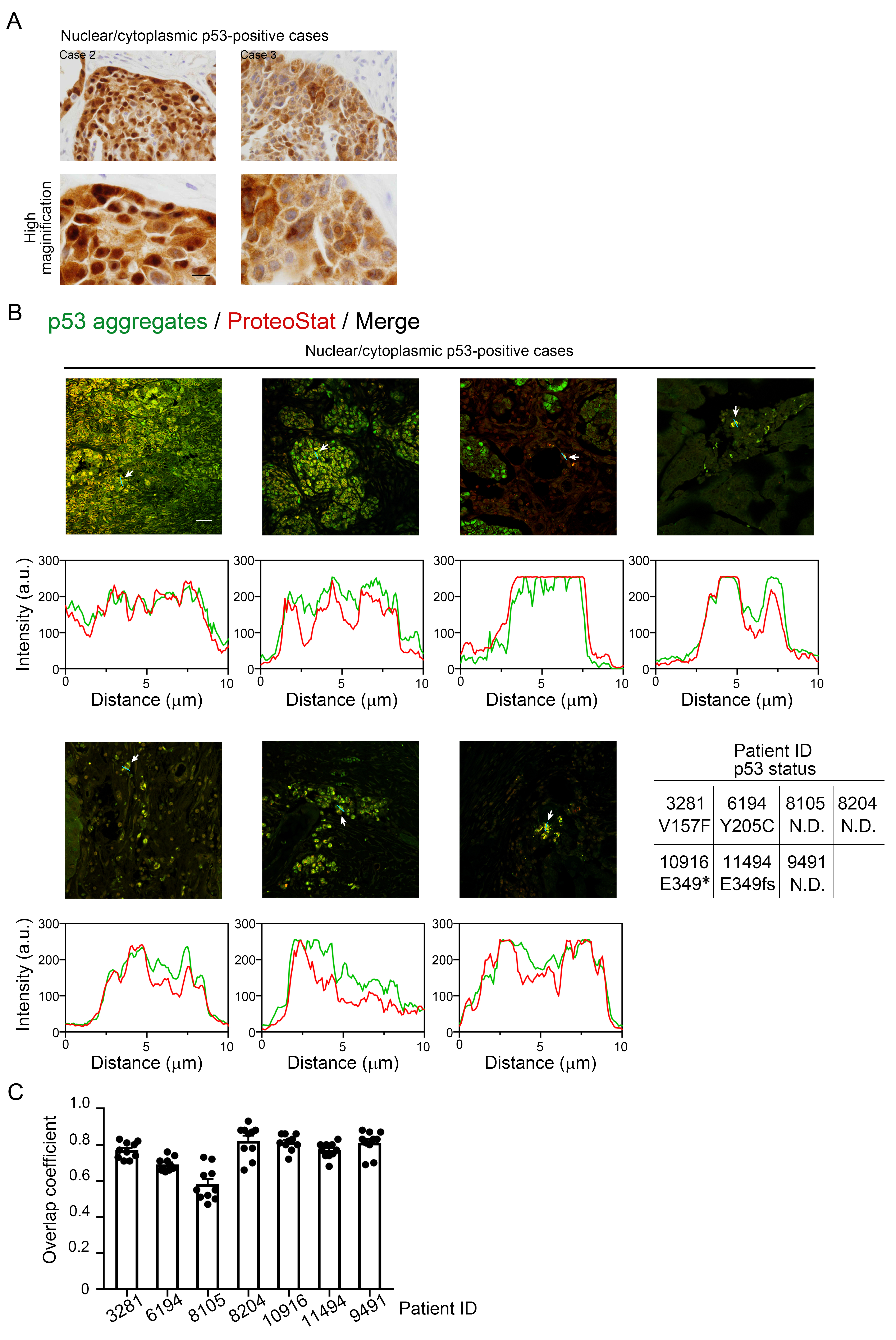


**Supplemental Figure S1 Immunohistochemical p53 (A) and ProteoStat (B) staining in nuclear/cytoplasmic p53-positive cases.**

**A** Additional immunohistochemical images of HGSOC tissues with nuclear/cytoplasmic p53-positive patterns. Scale bar: 20 µm. **B** Paraffin-embedded blocks of HGSOC tissues were cut into 3-µm-thick sections, followed by deparaffinization with xylene and rehydration with graded ethanol. Antigens were retrieved by using heat treatment, after which sections were blocked in Animal-Free Blocker and incubated overnight at 4°C with the E26 monoclonal anti-human p53 antibody, followed by an Alexa 488-conjugated polyclonal goat anti-rabbit IgG. Sections were then incubated in ProteoStat solution for 3 min, destained in 1% acetic acid for 20 min at room temperature, and mounted with Vectashield mounting medium with DAPI. The inset indicates the p53 status of each nuclear/cytoplasmic p53-positive case. N.D., not determined. The signal intensities along the line markers (blue lines) indicated by arrows in the images were measured for the green channel (p53) and the red channel (ProteoStat). Scale bar: 20 µm. **C** Quantification of colocalization of the green channel (p53) and the red channel (ProteoStat) obtained with Pearson’s cross-correlation coefficient analysis. Pearson’s cross-correlation coefficients were determined for 10 randomly selected regions of interest (20 µm × 20 µm) for each specimen.


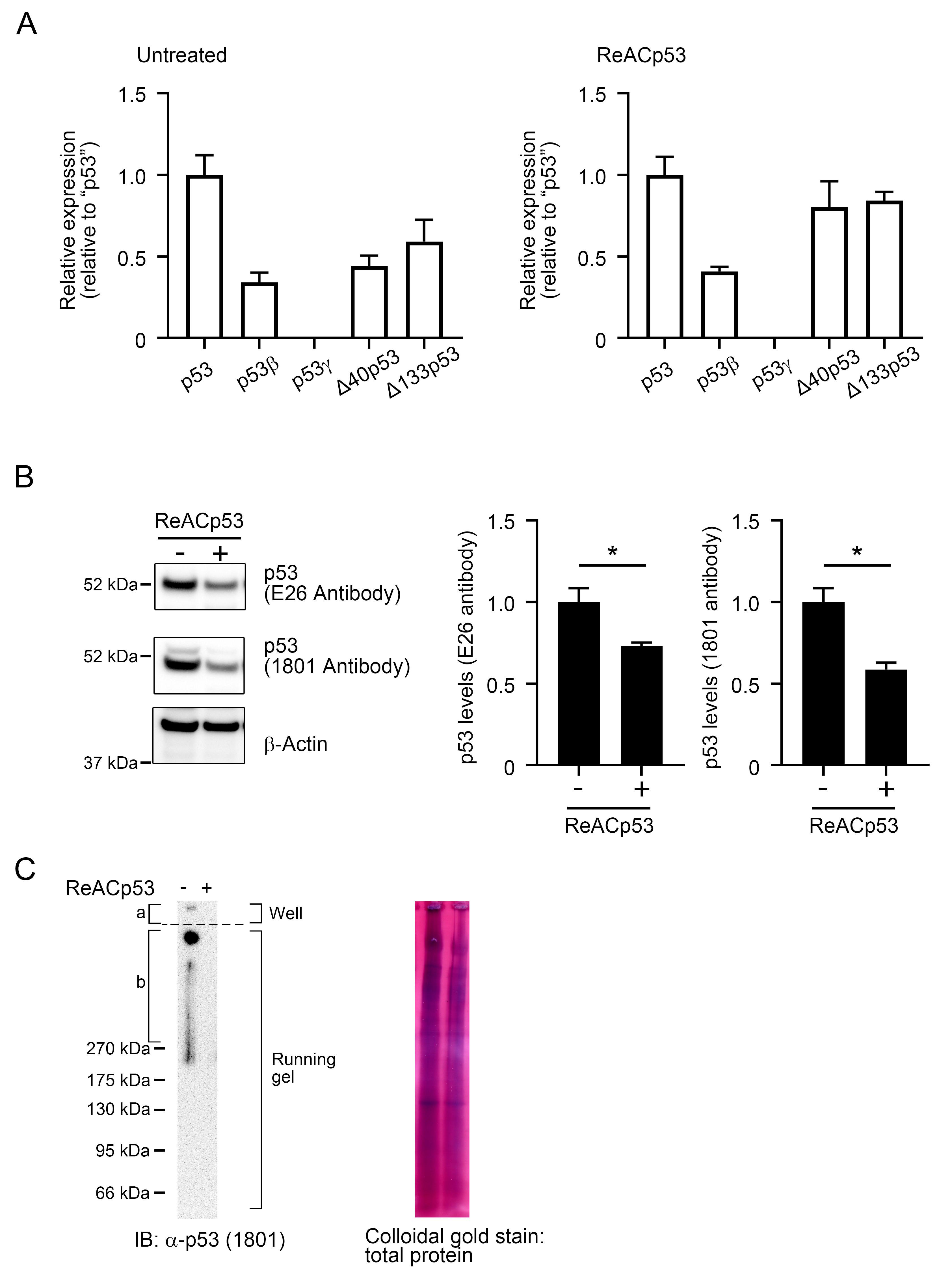


**Supplemental Figure S2 Transcriptional levels of p53 isoforms in OVCAR-3 cells.**

**A** Relative transcript levels of fl-p53 (p53), p53β, p53γ, Δ40p53, and Δ133p53 isoforms were determined by using RT-qPCR. The *GAPDH* gene was used for normalization. Data are means ± SE of three independent experiments. **B** Effects of ReACp53 (15 µM) on protein levels of p53 in OVCAR-3 cells were analyzed by means of Western blotting with the E26 and pAb1801 anti-p53 antibodies. *, *p* < 0.05 by the unpaired *t*-test. **C** Effects of ReACp53 (15 µM) on cytosolic p53 aggregates in OVCAR-3 cells were analyzed by means of BN-PAGE and Western blotting with the pAb1801 anti-p53 antibody. Large amyloid fibrils that did not enter the running gel (a) as well as many aggregated species with various molecular weights were observed (b).

**Supplemental Figure S3 p53 R248W‐transfectant PC‐3 cells extracellularly released OC‐ and A11-positive p53 aggregates.**

p53‐null PC‐3 cells were transfected with pCMV‐Neo‐Bam (mock) or pCMV‐Neo‐Bam p53 R248W. At 48 h after the transfection, conditioned media samples were collected, and protein aggregates were immunoprecipitated with the A11 anti-oligomer antibody or the OC anti-amyloid antibody. p53 protein levels in the immunoprecipitates were analyzed by means of Western blotting with the DO‐1 anti‐p53 antibody. IP, immunoprecipitation.

**Supplemental References**

1. Kobel M*, et al.* (2016) Optimized p53 immunohistochemistry is an accurate predictor of TP53 mutation in ovarian carcinoma. *J Pathol Clin Res* 2(4):247-258.

2. Matafora V*, et al.* (2020) Amyloid aggregates accumulate in melanoma metastasis modulating YAP activity. *EMBO Rep* 21(9):e50446.

3. Torres AJ, Vasudevan L, Holowka D, & Baird BA (2008) Focal adhesion proteins connect IgE receptors to the cytoskeleton as revealed by micropatterned ligand arrays. *Proceedings of the National Academy of Sciences of the United States of America* 105(45):17238-17244.

4. Singhai A*, et al.* (2014) Spatially defined EGF receptor activation reveals an F-actin-dependent phospho-Erk signaling complex. *Biophys J* 107(11):2639-2651.

5. Dunn KW, Kamocka MM, & McDonald JH (2011) A practical guide to evaluating colocalization in biological microscopy. *Am J Physiol Cell Physiol* 300(4):C723-742.

6. Melo Dos Santos N*, et al.* (2019) Loss of the p53 transactivation domain results in high amyloid aggregation of the Delta40p53 isoform in endometrial carcinoma cells. *J Biol Chem* 294(24):9430-9439.
